# Functional Quadriplegia as an Initial Presentation of Severe Rheumatoid Arthritis

**DOI:** 10.7759/cureus.33693

**Published:** 2023-01-12

**Authors:** Nadia G Obaed, Mohamed Elsheshtawi, Can Jones, Vivek Kothari, Tabitha Estica, Kristina Menchaca, Shaun Isaac

**Affiliations:** 1 Allopathic Medicine, Nova Southeastern University Dr. Kiran C. Patel College of Allopathic Medicine, Fort Lauderdale, USA; 2 Internal Medicine, University of Miami, John F. Kennedy (JFK) Medical Center, Atlantis, USA

**Keywords:** rheumatoid arthritis, atypical presentation, financial impact, economic burden of healthcare, diagnostic delay, limb edema, progressive weakness, functional quadriplegia

## Abstract

Rheumatoid arthritis is a chronic inflammatory condition with many manifestations primarily presenting in older female patients with joint stiffness. Quadriplegia associated with rheumatoid arthritis is common and can occur secondary to spinal cord compression from atlantoaxial dislocation. In contrast, functional quadriplegia is rare and has not been previously reported as an initial manifestation of rheumatoid arthritis. We report the case of a 56-year-old male with a past medical history of carotid artery stenosis, hypertension, and tobacco and alcohol misuse who presented to the emergency department with a five-month history of progressive bilateral shoulder pain and weakness resulting in functional quadriplegia. The patient required inpatient hospital admission for further evaluation of his functional quadriplegia and associated symptoms. His workup was significant for rheumatoid arthritis, and he was successfully treated with high-dose steroids and received physical and occupational therapy during admission. Prior to discharge, the patient was initiated on methotrexate therapy and appointed a follow-up with primary care and rheumatology. The purpose of this study is to facilitate early clinical recognition of a common disease with unique and underreported symptomatology.

## Introduction

Rheumatoid arthritis (RA) is a chronic autoimmune condition with multiple manifestations that can affect various parts of the body and significantly alter daily functional living when untreated. The prevalence of rheumatoid arthritis is approximately 1% worldwide and occurs more commonly in people aged 40 to 56 years old, predominantly affecting females [1,2]. The epidemiology of RA is also influenced by geographic location, race, genetics, and lifestyle factors. The most significant genetic risk factor for RA development is shared epitope alleles, while smoking is the strongest environmental risk factor for RA [3]. 

The etiology of RA remains unknown, but the disease process is dynamic. Immunological activation and inflammatory pathways have provided the framework for RA. Synovial hyperplasticity stemming from increased infiltration of immune and inflammatory cells, primarily fibroblasts and macrophages, contributes to the synovitis and joint destruction of RA [4]. The resulting synovial pannus of RA is accompanied by angiogenesis, eventually eroding cartilage and bone. More specifically, the degradation occurs through the cytokine production of Interleukin-1 (IL1) and tumor necrosis factor (TNF) alpha which inhibits new cartilage formation and promotes the differentiation of osteoclasts. 

The symptoms of RA fluctuate with flares and remission periods. Typically, RA symmetrically affects small peripheral joints that extend proximally with disease progression [5]. Morning stiffness for at least an hour or more, joint pain, and swelling are characteristic of RA [5]. Commonly seen extra-articular manifestations of RA include rheumatoid nodules, especially at pressure points, ulcerative skin lesions, vasculitis, Sjogren's syndrome, anemia of chronic disease, and pulmonary manifestations [6]. The presence of these extra-articular manifestations, as well as high rheumatoid factor titer,s correlates to disease severity and mortality [7,8]. 

Rarely, patients can present with axial involvement, and if so, the manifestation is usually secondary to an atlantoaxial lesion and confined to the cervical region [9]. There are limited reports of noncervical quadriplegia in association with RA and no reports specific to functional quadriplegia. According to the International Classification of Diseases-10, functional quadriplegia (FQ) is defined as the "complete immobility due to severe disability or frailty from another medical condition without injury to the brain or spinal cord" [10]. The purpose of this study is to facilitate the early clinical recognition of a common disease, RA, when a patient has unique and underreported symptomatology, including functional quadriplegia.

## Case presentation

A 56-year-old Caucasian male with a past medical history of bilateral carotid artery stenosis, hypertension, chronic alcohol use, and a 90-pack per year smoking history presented to the emergency department (ED) with four months of generalized weakness and a one-month history of functional quadriplegia and bilateral upper extremity swelling. The patient explained that his weakness gradually progressed from his left to right shoulder until he experienced immobility and constant dull pain in his bilateral upper extremities that radiated across his chest. He notes that he was unable to take care of himself during this period. Associated symptoms included fifteen-pound weight loss, decreased appetite, and bilateral lower extremity weakness. Given his recent weight loss, anemia, and progressive weakness, our initial differential included primary malignancy such as multiple myeloma or metastatic malignancy, paraneoplastic syndrome such as Lamber-Eaton syndrome, pernicious anemia, and inflammatory conditions such as systemic lupus erythematosus, Sjogren's syndrome, and rheumatoid arthritis. In the past month, it was revealed that he had received a blood transfusion for severe anemia and was worked up for malignancy which included an inconclusive lymph node biopsy and a negative bone marrow biopsy.

Physical examination demonstrated a pale, cachectic male appearing older than the stated age with fatigue and generalized pain. No obvious lymphadenopathy was noticed bilaterally. Musculoskeletal and extremity examination showed decreased range of motion and resistance to passive movement in bilateral upper and lower extremities, bilateral shoulder, hand, and knee tenderness upon palpation, bilateral upper extremity (UE) joint stiffness, and bilateral edema distal from his elbows to hands and right foot edema. Neurological examination showed grade 1 motor strength in bilateral UE, grade 2 motor strength in bilateral lower extremities (LE), and LE fasciculations. His skin examination revealed a stage 2 pressure ulcer at the right olecranon process. 

**Figure 1:**
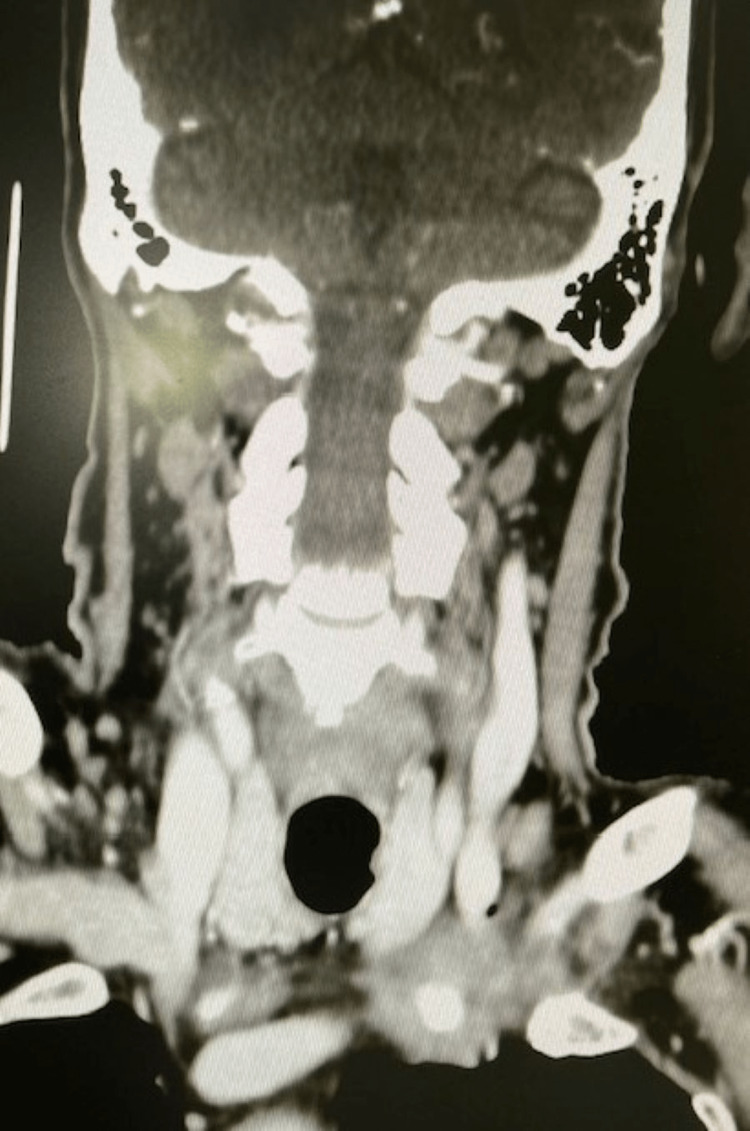
CT scan of the neck CT neck with contrast performed which detected no mass or lymphadenopathy

**Figure 2:**
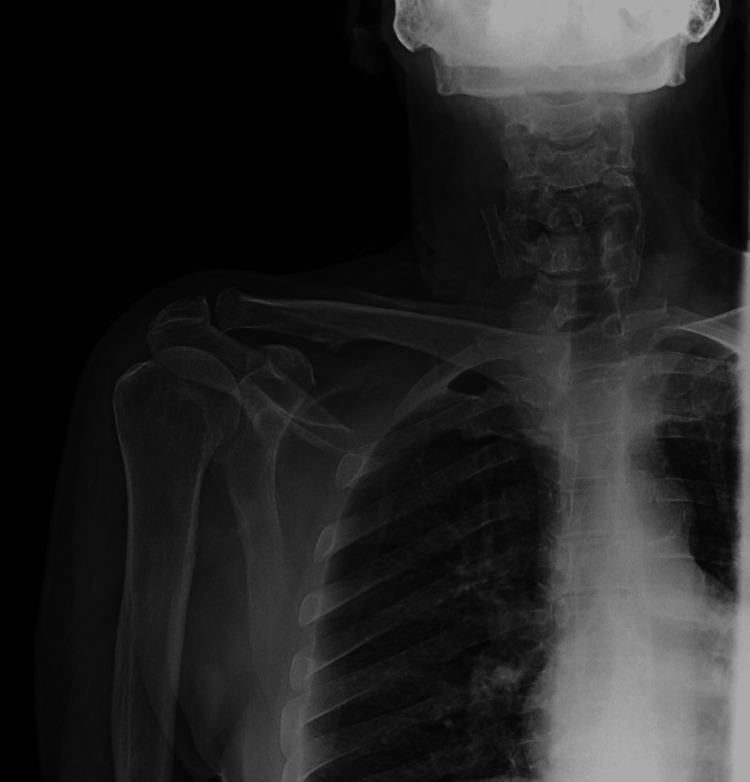
Shoulder X-ray Normal X-ray examination of the right shoulder, including normal glenohumeral articulation, acromioclavicular joint, acromion, and humeral head. The soft tissue structures are unremarkable. Normal visualized pulmonary apex.

The patient's initial laboratory assessment demonstrated normocytic anemia (mean corpuscular volume (MCV) of 88.7 and Hgb of 9.3 g/dL) and an elevated D-dimer (14,052 ng/mL), erythrocyte sedimentation rate (ESR) (114 mm/hr), C-reactive protein (13.1 mg/L), and creatine phosphokinase (155 mg/L) (Table 1). An initial imaging workup was completed to evaluate our initial differential diagnosis which included UE deep venous thrombosis, tumor mass specifically a Pancoast tumor or brain mass, pulmonary embolism, stroke, or rotator cuff injury. CT of the head, neck, and chest only corroborated the patient's medical history and did not show any new significant abnormalities (Figure 1). A bilateral shoulder X-ray and venous ultrasound (US) were normal (Figure 2). 

**Table 1:** Laboratory evaluation Select laboratory data from the patient's initial evaluation including complete blood count and complete metabolic panel. CO2 - carbon dioxide; BUN - blood urea nitrogen; AST/SGOT - aspartate aminotransferase/serum glutamic-oxaloacetic transaminase; ALT/SGPT - alanine aminotransferase/serum glutamic pyruvic transaminase; MCV - mean corpuscular volume; ESR - erythrocyte sedimentation rate.

Test	Patient’s result	Reference range	Units
Sodium	134	135-145	mmol/L
Potassium	4.3	3.5-5.2	mmol/L
Chloride	95	95-110	mmol/L
CO_2_	36	19-34	mmol/L
BUN	16	6-22	mg/dL
Creatinine serum	0.72	0.43-1.13	mg/dL
Calcium	10.0	8.4-10.2	mg/dL
Protein total	7.6	5.5-8.7	g/dL
Albumin	3.5	3.2-5.0	g/L
Magnesium	2.1	1.6-2.4	mg/dL
Alkaline Phosphatase	95	30-130	IU/L
AST/SGOT	29	<41	IU/L
ALT/SGPT	24	<41	IU/L
Bilirubin-total	0.4	0.1-1.2	mg/dL
C-reactive protein	13.1	<10.0	mg/L
Glucose	166	70-110	mg/dL
Creatine phosphokinase	155	10-120	mcg/L
Troponin 1 cardiac	<0.012	0.000-0.034	ng/mL
Red blood cell count	3.36 x 10^6^	4.63-6.08 x 10^6^	uL
Hemoglobin	9.3	13.7-17.5	g/dL
Hematocrit	29.8	40.1-51.0	%
White blood cells	11.2 x10^3^	4.0-10.5 x10^3^	u
Platelets	492 x10^3^	150-400 x10^3^	uL
MCV	88.7	79.0-92.2	fl
D-dimer	14,052	<500	ng/mL
ESR	114	0-22	mm/hr

Follow-up evaluation with a hypercoagulable panel was within normal limits, including protein C, anti-Mi-2, and anti-synthetase. However, the rheumatologic workup showed an elevated cyclic citrullinated peptide (>300 u/mL) and rheumatoid factor (266.8 IU/mL). The patient was diagnosed with functional quadriplegia secondary to previously undiagnosed rheumatoid arthritis. He was successfully treated, given his significant improvement in physical mobility, with high-dose prednisone. The patient had minimal tenderness in the upper extremities, increased range of motion with minor difficulty reaching full shoulder flexion, and no resistance in passive movement. He also had a full active range of motion in the lower extremities. Furthermore, the patient received physical and occupational therapy during admission. Prior to discharging the patient to his home, he was initiated on methotrexate therapy, appointed a follow-up with primary care and rheumatology, and given a short course of steroids to continue at home.

## Discussion

The patient presentation in our case is unique as there are no other reports in the literature describing functional quadriplegia as the initial presentation of RA. While RA most commonly presents with peripheral joint stiffness and pain in the hands, our patient demonstrated functional quadriplegia with significant associated bilateral upper extremity swelling that significantly and rapidly improved with treatment. His symptoms which began as inflammatory, potentially RA related, had gradually progressed over a total five-month period to complete immobility, and he had been seen by other physicians prior to arrival at our ED without a proper diagnosis. Furthermore, the criteria for RA diagnosis is not well established as there is no single pathognomonic test for RA. Thus, we are highlighting the importance of acknowledging RA as part of a broad differential when evaluating for functional quadriplegia. The threshold for suspecting RA and ordering a rheumatologic workup should be low in the presence of progressive weakness and extremity pain in a middle-aged male with strong risk factors known to be associated with RA. 

Functional quadriplegia is usually associated with neurologic pathologies including end-stage dementia, advanced multiple sclerosis, and amyotrophic lateral sclerosis [11]. Although, anyone with the inability to move their extremities in the absence of neurologic injury that also requires full assistance with activities of daily living should be assessed as functionally quadriplegic. There is very limited research in terms of describing and evaluating FQ, but this report supports the inclusion of musculoskeletal and joint disorders as part of the initial workup. A nationwide analysis conducted between 2009 to 2014 showed that FQ had an exponentially rising incidence, a 41% increase in length of stay, and 17% higher hospital charges [12]. Given that the population most affected by FQ tend to be elderly patients with other comorbidities, an earlier discharge to better equipped facilities such as long-term acute care hospitals (LTACH) can help to not only curb cost, but also reduce patient exposure to hospital acquired conditions. Furthermore, the increased hospital resource utilization of FQ can be mitigated with proper early and complete evaluation for a reversable FQ etiology. The study also revealed that most patients diagnosed with FQ had Medicare highlighting the necessity for promoting health literacy and advocating for primary care follow-up among our elderly population [12]. 

The patient in our case report also did not regularly follow up with a primary care physician which most likely contributed to both the delayed diagnosis and the severity of RA at time of arrival. Given that this patient had Medicaid insurance, we investigated the associated cost of RA in similarly insured patients. One cost-of-care analysis included three studies for patients enrolled in Medicaid in which the average age of analyzed patients was 50 years old from a combined total of 9,064 patients [13]. The annual incurred cost of RA-specific care estimate among all included studies was lowest for Medicaid patients, ranging from $3,486 USD to $7,489 USD, and from $3,266 USD to $19,519 USD for total cost of care [14-16]. Although the cost seems low, RA-specific costs take up at least 38% of total cost of care [13]. Moreover, these studies are not restricted to patients using the standard of care for RA which is conventional disease modifying anti-rheumatic drugs (DMARDs), and in case of failure, their alternative, biologic DMARDs. This can significantly skew costs of RA care when the majority of patients in the study utilize narcotic analgesics, non-steroidal anti-inflammatory medication, and oral steroids [13]. Prescription claims for either DMARDs or biologic DMARDs are expected to greatly increase the financial burden of RA. Another limitation of all studies included in the meta-analysis is that they are based on costs dated prior to 2010, calling for a more updated cost-of-care analysis to accurately reflect the changing healthcare costs in recent years [13]. Delayed diagnosis, such as in our patient secondary to an atypical manifestation with FQ, increases both the rate of health care service utilization and the cost to the patient. The prevalence of musculoskeletal conditions and arthritis, especially when there is associated disability, were deemed to have the highest impact on the healthcare system. According to the National Medical Expenditure Panel Survey (MEPS) data, the economic burden is estimated at $128 billion USD with $80.8 billion USD attributed to direct medical expenditures and the remaining to indirectly lost earnings [17]. These indirectly lost earnings are surmised through lost wages and productivity. 

In regard to direct patient health, a later diagnosis produces a higher risk of disability and death [18]. Joint pain is the leading symptom prior to RA diagnosis at 94% [19]. Our patient's symptomatology also included joint pain, but most noticeably was his immobility which was an unreported manifestation according to a study investigating diagnostic delays associated with arthritis. Other symptoms that coincided with our patient were reported such as joint swelling (78%), fatigue (76%), and no appetite or unintentional weight loss (39%) [19]. The most cited factors for delayed physician visits were limited access to specialists, conviction that symptoms will self-resolve, and unaffordable free time for a visit [19]. Therefore, the diagnostic delays can be derived from ease of access to healthcare, health literacy, the patient's insurance, and socioeconomic status. Although our patient had no primary care follow-up, he had been to a hospital in the month prior to his initial presentation. Thus, diagnostic accuracy and physician evaluation can also be implicated. Organizational changes in healthcare such as direct referral to a rheumatologist can limit diagnostic delay. Physicians may experience cognitive bias as they reflexively develop a heuristic for evaluating patients with FQ for etiologies involving the nervous system including neoplasms without including more common comorbidities like musculoskeletal diseases. 

## Conclusions

Overall, our case study demonstrates an atypical initial presentation of RA in which a middle-aged Caucasian male without proper primary care follow-up presented with functional quadriplegia and upper extremity edema causing severe disability in activities of daily living. This report emphasizes the broad differential that accompanies weakness and functional quadriplegia. Most profoundly, the study critically appraises the consequence of an incomplete patient workup rendering the progression of a debilitating disease with an analysis of the concomitant economic expenditure. 
